# Technological Aspects of Chemoenzymatic Epoxidation of Fatty Acids, Fatty Acid Esters and Vegetable Oils: A Review

**DOI:** 10.3390/molecules201219778

**Published:** 2015-12-02

**Authors:** Eugeniusz Milchert, Kornelia Malarczyk, Marlena Kłos

**Affiliations:** Institute of Organic Chemical Technology, Faculty of Chemical Engineering, West Pomeranian University of Technology Szczecin, 10 Pulaski St., 70-322 Szczecin, Poland; Kornelia.Malarczyk@zut.edu.pl (K.M.); Marlena.Klos@zut.edu.pl (M.K.)

**Keywords:** fatty acids, alkyl fatty acid esters, free fatty acids, epoxidation

## Abstract

The general subject of the review is analysis of the effect of technological parameters on the chemoenzymatic epoxidation processes of vegetable oils, fatty acids and alkyl esters of fatty acids. The technological parameters considered include temperature, concentration, amount of hydrogen peroxide relative to the number of unsaturated bonds, the amounts of enzyme catalysts, presence of solvent and amount of free fatty acids. Also chemical reactions accompanying the technological processes are discussed together with different technological options and significance of the products obtained.

## 1. Introduction

Epoxidized vegetable oils and the products of their transformations—fatty acids and alkyl esters of fatty acids—are in widespread use. Epoxidized vegetable oils are used as solvents [1], lubricants [2,3,4,5], prepolymers in surface coating formulations [6,7,8,9], stabilizers (PVC) and plastics plasticizers [10,11,12,13,14,15], asphalt additives and transformer fluids [16]. They are used for production of polyetherpolyols and polyesterpolyols as intermediates in polyurethane production [14,15,17], block copolymers in polyester resin production [18], and nanocomposites. Using epoxidized oils it is possible to obtain polymers and composites with better mechanical [19,20,21], electric [22], thermal [23] properties than those of the polymers obtained from petrochemical products [20,22] and greater resistance to oxidation [22,23] than the latter ones. Epoxidized oils are auxiliary agents used to improve the efficiency of linoleum flooring production and modification of other thermoset polymers [24,25,26]. They are important intermediates in organic synthesis as they participate in many reactions thanks to the high reactivity of oxirane rings [27].

## 2. The Methods for Epoxidation of Vegetable Oils

There are a few industrially important vegetable oil epoxidation methods:

- Reaction with pre-produced peroxyacids or generated *in situ* in acidic homogeneous medium [23,28,29,30,31,32],
- Epoxidation with peroxyacids in the presence of cationic ion-exchange resins (Amberlite^®^ IR-122, Amberlite IR 120H, KU-2 × 8) [33,34,35,36,37,38,39,40,41,42,43,44,45],
- Epoxidation over phosphotungstate heteropolyacid catalysts and in the presence of phase-transfer catalysts (H^+^/WO_4_^−2^/PO_4_^−3^/Q^+^X^−^), QX-onium salt [42,43],
- Epoxidation over titanium-silicalite catalysts (Ti(IV)-SiO_2_, Ti-MCM-41, and amorphous Ti/SiO_2_) [39,44,46,47],
- Epoxidation in the presence of transition metal complexes as catalysts (CH_3_ReO_3_ or CH_3_ReO_3_/Nb) [48,49],
- Epoxidation in the presence of aluminium trioxide, obtained by a sol-gel method [50],
- Chemoenzymatic epoxidation [28,51,52,53,54,55].

The first of the abovementioned methods is used for the industrial scale production of soybean and rapeseed oils. In 2013 the production reached more than 200,000 metric tons per year of epoxidized soybean oil. The yields of the processes are different. Not all oils can be epoxidized using peracids (tung oil, for example) which is related to the content and type of unsaturated acids in triglycerides. Other vegetable oils that can be epoxidized include: linseed, corn, safflower, melon seed, cotton seed [56], rubber seed oil [57,58]. Epoxidations of inedible vegetable oils like canola [36], mahua [28], jatropha [38], and karanja [34] have been reported.

## 3. Reactions of Chemoenzymatic Epoxidation

**Scheme 1:**
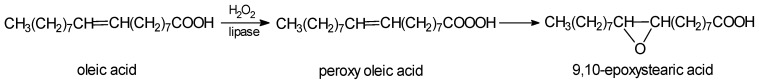
Chemo-enzymatic self-epoxidation of unsaturated fatty acids: conversion of oleic acid to epoxystearic acid.

**Scheme 2:**
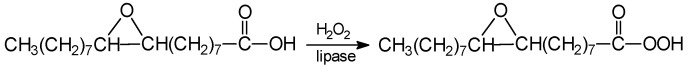
Formation of epoxidized peroxy fatty acids.

**Scheme 3:**
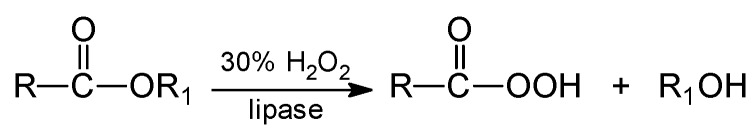
The reaction of carboxylic acids esters with hydrogen peroxide.

**Scheme 4:**
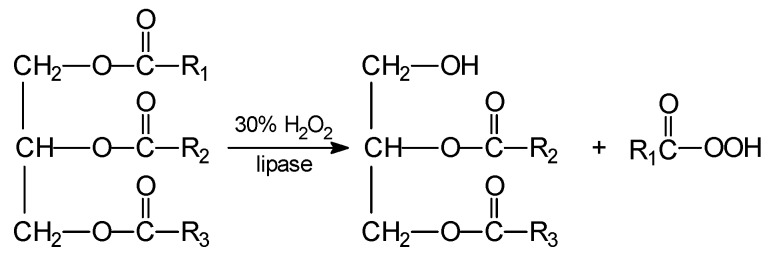
Perhydrolysis of triglycerols.

Chemoenzymatic epoxidation is performed in the presence of *Candida antarctica* lipase B (CALB), CALB immobilized onto an acrylic resin (Novozym^®^435) or *Candida antarctica* lipase B immobilized onto silica (CALB-silica) and another lipases as catalysts. Lipases can also catalyse the hydrolysis of triglycerides, alcoholysis, acidolysis, esterification, interesterification and aminolysis. In the presence of hydrogen peroxide and lipase unsaturated and/or saturated fatty acids are transformed into peroxyacids. Novozym^®^435 shows a high enzymatic activity among the hitherto examined enzymes [57]. The actual epoxidizing agent is the peroxyacid, while carboxylic acid acts as oxygen carriers. Unsaturated peroxyacids can also undergo self-epoxidation, according to the reaction [51,52,53,54,59] shown in Scheme 1:

In this case unsaturated fatty acids are converted into unsaturated peroxyacids thanks to the perhydrolysis activity of lipase, and then unsaturated peroxy or carboxylic acids are epoxidized via the classical uncatalyzed reaction referred to as self-epoxidation. In fact it proceeds mostly via an intermolecular process [53]. Epoxidized fatty acid can also form epoxidized peroxy fatty acids, which are also epoxidizing agents (Scheme 2):

The alkyl esters of carboxylic acids, in the presence of enzyme lipase and hydrogen peroxide, undergo perhydrolysis with formation of the corresponding peroxy acids (Scheme 3): 

Glycerides undergo perhydrolysis in a similar way (Scheme 4):

Lipase B shows lower catalytic activity in the epoxidation of glycerides than in the process with alkyl esters of monocarboxylic acids [58]. The low hydrolytic activity towards triglycerides of long chain fatty acids is probably due to the narrow and deeply located active sites of the enzyme [60,61]. Lipase B are also the catalyst of triglycerides transesterification and hydrolysis. The product of epoxidation of oils containing unsaturated fatty acids is a mixture of epoxidized mono-, di-, triglycerides, glycerol and epoxidized free fatty acids [51,62,63].

Epoxidation of rapeseed, sunflower and linseed oils involves a maximum of 88%–95% of C=C bonds [51,52,53,54,63]. Perhydrolysis of oils leads to the formation of peroxy fatty acids and mono- and diglycerides. Their removal is practically impossible. The addition of free fatty acids (in a maximum amount up to 5% with respect to C=C bonds in a given oil) to a given oil prevents or substantially restricts the amount of mono- and diglycerides. Perhydrolysis occurs, but the hydroxyl groups of glycerol, mono- and diglycerides are reesterified by the excess of free fatty acids. In this way, the reaction product contains only epoxidized triglycerides and epoxidized free fatty acids. Free fatty acids can be easily removed by alkaline washing. In most applications the presence of these by-products does not matter. Their amount in the post-reaction mixture is determined by the equilibrium of the three simultaneous reactions:

- hydrolysis of ester to alcohol and free acid,
- perhydrolysis of ester with hydrogen peroxide to alcohol and peroxy acid,
- oxidation of free fatty acid with hydrogen peroxide to peroxy acid.

Chemoenzymatic epoxidation is characterised by high chemo-, regio- and stereoselectivity [16]. Lipase immobilized on the adsorbent can be easily recycled.

## 4. Technological Parameters of Chemoenzymatic Epoxidation

A typical reaction system for a biocatalytic reaction is composed of the water layer containing hydrogen peroxide, organic layer containing a solvent, usually toluene, a vegetable oil or its alkyl ester and immobilised enzyme (solid phase) [26]. Chemoenzymatic epoxidation of soybean, rapeseed, linseed, corn, sunflower and olive oils has been best described. There is also substantial information on the epoxidation of oleic, linoleic, linolenic, erucic acids and alkyl esters thereof. 

### 4.1. Amount of Hydrogen Peroxide and Time of Reaction 

Orellana-Coca *et al.* have reported that the amount of hydrogen peroxide has the greatest influence on the rate of the reaction and the degree of epoxidation (conversion) [64,65]. An excess of hydrogen peroxide relative to the number of unsaturated bonds is necessary. This permits reaching full conversion of double bonds and compensation of hydrogen peroxide loss, caused by its decomposition at temperatures above 50 °C. The challenge is to shorten the reaction time. Too long a reaction time (6 to 12 h) and excess of hydrogen peroxide lead to increased level of carboxylic peracids in the final product. Peracids could be a potential problem for reasons of safety and contamination of the final product. The reaction rate increased with increasing hydrogen peroxide concentration between 10–50 wt %. However, it was accompanied with the enzyme inactivation. Linoleic acid was completely epoxidized when used at a concentration of 0.5–2.0 M in toluene at 30 °C. In a solvent free medium the reaction was incomplete due to the formation of a solid or a highly viscous oily phase, which increased the resistance to mass transfer.

### 4.2. The Effect of Process Temperature and Type of Solvent

An increase in temperature to a value not exceeding 50 °C also increases the rate of the reaction because it does not lead to hydrogen peroxide decomposition and enzyme inactivation. Tőrnvall *et al.* have performed chemoenzymatic epoxidations of unsaturated oleic and perpalmitic fatty acids in the presence of immobilized *Candida antarctica* lipase B [66]. Lipase B has been immobilized onto acrylic resin, in the presence of different solvents: toluene, water, H_2_O_2_, oleic acid, perpalmitic acid and epoxystearic acid, in the temperature range 20–60 °C, and then its catalytic activity in the process of epoxidation was evaluated. Epoxystearic acid caused a slight enzyme deactivation at 50 °C, in contrast to oleic acid and perpalmitic acid. Also a mixture of toluene and water did not cause deactivation of lipase B in the range 20–60 °C and in a 48 h period. The activity of lipase B was maintained in the presence of 6–12 M hydrogen peroxide at 20 °C, but at 60 °C it rapidly decreased. Also with increasing concentration of hydrogen peroxide the rate of lipase deactivation increased. Gradual addition of lipase B was particularly important for the processes carried out in higher temperatures and at the beginning of the process, before the water generated in the process could dilute the hydrogen peroxide. The activity of lipase decreased as a result of a too high concentration of H_2_O_2_ and a too high temperature (60 °C). Because of the exothermic character of epoxidation, this fact is of particular concern for the processes carried out on large scale. Toluene is the best stabiliser of lipase and it facilitates contact between the substrates and biocatalyst.

In the presence of catalytic amounts of lipase B, simultaneous esterification and epoxidation of oleic acid was also performed [67]. Higher degrees of esterification and epoxidation were obtained with *n*-octanol, *n-*hexanol, n-butanol than with ethanol and 2-propanol. The rate and yield of epoxidation were the highest in the reaction with 2-propyl oleate and lower with other alcohol esters. The esterification was relatively fast, like epoxidation of oleic acid and co-occurring alkyl ester. The main problem with the use of lipase B was a considerable decrease in its activity caused by the presence of concentrated hydrogen peroxide. Gradual addition of hydrogen peroxide also reduced the enzyme deactivation. The decrease in lipase B activity caused by the presence of alcohols was smaller than that caused by hydrogen peroxide, and the greatest in the presence of ethanol. Enzymatic synthesis of alkyl epoxystearate with employment of oleic acid is simpler and more energy saving than the classical method of epoxidation with peroxy acids (performic or peracetic acid). The former process does not need the use of additional solvents so the isolation of the main product is easier. High yields of epoxystearic acid and epoxystearic acid methyl ester have also been obtained by epoxidation of oleic acid or its methyl ester under solvent free conditions [68]. Bjőrkling *et al.* have confirmed a decrease in lipase B activity after a few cycles of epoxidation of alkenes [69]. In nonpolar solvents, toluene and hexane, this decrease was much slower.

High yield and rate of the reaction have been reported by Silva *et al.* in epoxidation of methyl oleate in the presence of hydrophilic ionic liquid and *Aspergillus niger* lipase [70]. A hydrophilic ionic liquid, butylmethylimidazolium tetrafluoroborate [bmi][BF_4_], allowed production of the epoxidized compound in 89% yield after the first hour of reaction. In the presence of hydrophobic butylmethyl-imidazolium hexafluorophosphate [bmi][PF_6_] the yield was 67%.

By enzymatic method, using the CALB catalyst immobilized on silica, excellent yields and selectivities of *cis*-methyl-9,10-epoxystearate were obtained by the epoxidation of methyl oleate [71]. The same yields were also obtained after a shorter reaction time as compared with the catalyst Novozym^®^435. However, the shortest reaction time were obtained during the epoxidation with hydrogen peroxide in the presence of bis-[3,5-bis(trifluoromethyl)diphenyl] as catalyst and 1,1,1,3,3,3-hexafluoroisopropanol as solvent (a nonenzymatic process). The shortest reaction time were obtained, but during the epoxidation with hydrogen peroxide in the presence of bis-[3,5-bis(trifluoromethyl)diphenyl] as catalyst and 1,1,1,3,3,3-hexafluoroisopropanol as solvent (nonenzymatic process).

### 4.3. The Presence of Free Fatty Acids in Oils or Fatty Acids Esters

Lu *et al.* have reported the highest epoxy oxygen group content in the epoxidation of soybean oil methyl esters in the presence of behenic and stearic acid [72]. The reaction was conducted at 0.001:1 mol/g ratio of free fatty acids to soybean oil methyl esters at 55 °C and 800 rpm mixing speed. The input material was composed of 10 g esters, 0.3 g lipase, 50 g toluene, 14 g H_2_O_2_ (35% concentration). For saturated and monounsaturated (C_12_-C_18_) free fatty acids (FFA), the epoxy oxygen content increased with increasing carbon chain length of FFA. For branched-chain unsaturated FFA, the epoxy oxygen group content decreased in the presence of hydroperoxide and hydroxyl group of FFA. The epoxy oxygen group content also decreased with increasing number of double bonds in FFA.

The optimum parameters of the process of chemoenzymatic epoxidation of soybean oil in the presence of lipase B immobilised on acryl resin have been established by Vlček and Petrović [73]. They used soybean oil containing 8 wt % of oleic acid and they changed the lipase concentration in the range 2.1–20.8 wt % relative to the oil content. A 35% solution of H_2_O_2_ was introduced into a mixture of soybean oil, toluene and lipase (molar ratio H_2_O_2_/C=C = 2:1). The number of unsaturated bonds in the soybean oil molecule was 4.5 mol/mol. After increasing the lipase concentration to 4 wt % relative to the oil content, a fast increase in the conversion of unsaturated bonds with formation of epoxy groups took place. The concentration of lipase significantly affects the formation of epoxide groups. Having increased lipase B concentration to 20 wt %, the conversion reached a stable level of 95–99 wt %, after 4 h at 50 °C. Conversion at a level of 40% was reached after 2 h. Addition of free fatty acids was not necessary as they were present in soybean oil as a result of partial hydrolysis of ester bonds. The conversion of soybean oil at the level 80% was commonly achieved in the system without the presence of any fatty acid.

Sun *et al.* have established the optimum technological parameters of corn oil epoxidation using H_2_O_2_ as an oxygen donor and stearic acid as an active oxygen carrier [74]. They obtained a relative conversion to oxirane of 85.3% and epoxy oxygen group content 5.8%. The optimum parameters found by these authors were: temperature 35 °C, amount of stearic acid 28% in relation to corn oil, mole ratio of H_2_O_2_/C=C bonds 2.7:1, reaction time 10 h. The impact of these parameters decreased in the order: amount of stearic acid > temperature ≈ molar ratio of H_2_O_2_/C=C bonds > reaction time.

Sun *et al.* have studied also chemoenzymatic epoxidation of *Sapindus mukorossi* seed oil [75]*.* Hydrogen peroxide and stearic acid were used as epoxidizing agents. The iodine number of the oil was 84.8 g/100g and the oil contained [wt %]: oleic acid 51.0, linoleic acid 6.6, linolenic acid 1.1 and eicosanoic acid 23.1. These authors showed that stearic acid enhanced the enzymatic epoxidation of oil. The content of epoxy oxygen groups in the obtained product was 4.6%. The optimised epoxidation conditions were as follows: temperature 50 °C, 4:1 molar ratio of H_2_O_2_/C=C, reaction time 7.0 h, the amount of lipase B 2.0 wt % relative to the oil weight. Relative conversion to oxirane was 90.2%. It results from these studies that the epoxidation of unsaturated fatty acids with hydrogen peroxide in the presence of simple carboxylic acids as well as by chemoenzymatic method proceeds faster than the epoxidation of vegetable oils. These research indicates that the epoxidation of unsaturated fatty acids both by the use hydrogen peroxide in the presence of simple carboxylic acids and chemoenzymatic are rapidly than the epoxidation of vegetable oils.

Schneider *et al.* have reported the optimal parameters of epoxidation of methyl esters of sunflower oil with lipase B and aqueous H_2_O_2_ in the presence and absence of an acyl donor (octanoic acid) [76]. The biphasic system CH_2_Cl_2_/H_2_O comprised 30% hydrogen peroxide and lipase B. The use of biphasic system for reduction of lipase inactivation was proposed as an alternative solution, because the enzyme remains protected in the aqueous phase. This process begins with perhydrolysis of methyl esters derived from sunflower oil, under mild conditions, catalysed by lipase. The conversion of unsaturated bonds reaches 99% both in the biphasic (CH_2_Cl_2_/H_2_O and monophasic (CH_2_Cl_2_) systems. The presence of caprylic acid as a peracids precursor is of crucial importance. The main products are mono- and diepoxystearates. The best technological parameters were found to be as follows: 30 °C, reaction time 16 h, 10 mmol octanoic acid in relation to 1 g of the oil, 1 mL 30% H_2_O_2_ in relation to 1 g of the oil, 100 mg lipase B, 6 mL CH_2_Cl_2_ and 5 ml of water. Lipase B was reused 10 times in successive experiments at 24 h intervals, without a significant loss of activity.

### 4.4. Effect of Simultaneous Changes in a Few Parameters

Chemoenzymatic epoxidation of linseed oil to obtain epoxyglycerides of different content of oxygen has been studied by López Téllez *et al.* [77]. On changing the temperature, amount of hydrogen peroxide of 30 or 50 wt % concentration and the amount of lipase B, the epoxidized oils demonstrated epoxidation degrees ranging from 8% to 54%. The absence of side reactions was confirmed by FTIR and Raman spectroscopy. Analysis of ^1^H-NMR data provided a linear relation between the number of double bonds and the epoxide number. 

Epoxidation reactions obtaining methyl epoxyricinoleate in the presence of lipase B have been studied by Kazariya and Matsumura for [78]. Lipase and hydrogen peroxide (30 wt %) were added to a solution of methyl ricinoleate in toluene, and the mixture was stirred at room temperature for 24 h. After separation, methyl epoxyricinoleate was polycondensed to polyepoxyricinoleate with an average molecular weight M_w_ = 272,000.

Besides epoxidation of fatty acids and their derivatives, lipase was used for epoxidation of phenolic compounds for the synthesis of phenolic epoxy prepolymers (Aouf *et al.* [79]). Urea hydrogen peroxide was used instead of hydrogen peroxide, and no significant differences in deactivation of lipase B were observed [67].

De Abreu Corrêa *et al.* have reported the following optimum parameters of epoxidation of oleic acid catalysed by PSCI-Amano lipase from *Burkholderia cepacia* immobilized on ceramic: 55 °C, 10% content of enzyme, 0.2% concentration of hydrogen peroxide, reaction time 3 h, stirring speed 150 rpm [27]. Under these conditions the epoxidation yield was close to 88%, while the conversion and selectivity of epoxidation of rapeseed, sunflower, soybean and linseed oils reached over 90% [52,53]. In the process of epoxidation of linseed oil, the content of oxirane oxygen was 9.9%, which is close to the theoretical value. High content of oxirane oxygen is especially desirable when the oil is to be used as a plasticizer and plastic stabilizer. When epoxidized oils are used as paint and varnish solvents, it is important that they show low viscosity, appropriate iodine number and oxirane oxygen contents. These properties should be similar to those found in natural oils containing epoxy groups, such as vernonia oil (viscosity 112 mP). A product of similar properties was obtained as a result of epoxidation of a mixture of sunflower and linseed oil (molar ratio 3:1). At a conversion of 40%, the epoxidized synthetic oil obtained showed properties closest to those of the natural vernonia oil (similar content of oxirane oxygen, epoxide number and viscosity). The difference between the two oils was in chemical structure; the synthetic product did not contain vernolic acid whose presence determined the oil properties and had a different spatial structure.

Chemoenzymatic epoxidation of linseed oil in a recirculating reactor with separation of immobilized lipase B from the water solution of hydrogen peroxide, has been reported by Hilker *et al.* [26]. This solution permitted a reduction of the deactivation of immobilized lipase dispersed within the organic phase, as the lipase contact with the aqueous hydrogen peroxide solution was limited. In order to achieve saturation with H_2_O_2_, the organic layer was passed through the aqueous layer containing H_2_O_2_ in a bubble column. The membrane was quickly flushed by reversing the direction of the pump to prevent blocking. The actual epoxidizing agent was perstearic acid. On the basis of kinetic studies it was determined that the stability and activity of the enzyme depended mainly on the reaction temperature and H_2_O_2_ concentration. 

## 5. Stability and Inactivation of Lipase B

Deactivation of lipase is the main problem in chemoenzymatic epoxidation of unsaturated compounds. High price and limited lipase use time make the process and its applications less economically advantageous than other types of epoxidation processes, especially the *in situ* peracid epoxidation. In batch processes the number of recirculations of a catalyst is also limited by the impossibility of catalyst regeneration. Warwel *et al.* have reported that on epoxidation of unsaturated fatty acids the were able to extend the lipase activity up to 15 cycles thanks to the use of toluene as a solvent [54]. The duration of one cycle was 8 h. Unfortunately, the lipase activity decreased to 75% of the initial one. To increase the lipase stability and number of use cycles, an excess of unsaturated compound with respect to hydrogen peroxide was tested. Hydrophobic solvents are generally preferred for lipase catalysed reactions. The negative influence of solvent polarity on lipase activity and stability seems indeed to be due to competition between the solvent and enzymatic protein for water [80]. Such a competition leads to the state of the protein hydration which is crucial for its activity. Hydrophobic solvents are not always used for lipase catalysed reactions involving polar substrates such as phenolic compounds or amino acids [81]. Solvents with intermediate polarities are also used like for example: *tert*-butyl alcohol [82], acetone [83], 2-methyl-2-butanol [84], methylene chloride [76], *etc*.

Decreases in lipase B activity in subsequent alkene epoxidation processes have also been reported by other authors [69]. The decrease was the slowest in the presence of nonpolar solvents, toluene and hexane. Optimisation of the technological parameters of epoxidation permitted extension of the time of lipase activity and its use [68]. The greatest impact on reduction of lipase stability and activity has a high concentration of hydrogen peroxide (above 12 M at 20 °C) and temperatures above 50 °C [66]. For this reason, diluted solutions of H_2_O_2_ are used and these solutions are dosed slowly. Water is also a good solvent for lipases. In biphasic aqueous-hydrophobic media this property allows one to increase the yield of epoxide. In this way at least one of the reactants is mainly water soluble and another one is hydrophobic. This allows easier solubilisation of polar and nonpolar reactants [81,82]. The most important technological parameters of chemoenzymatic epoxidation of vegetable oils, free fatty acids and their esters are presented in Table 1.

## 6. Conclusions

The only method of epoxidation applied on the industrial scale is that employing peracids either prepared in a separate step or generated *in situ*. Because of the risks related to handling peracids, the *in situ* method is preferred for industrial scale epoxidation of triglycerides [52]. In chemoenzymatic epoxidation the highest yields and transformation selectivities to epoxidized vegetable oils have been achieved in the presence of *Candida antarctica* lipase B. Although other enzymes have been also found able to catalyse epoxidations, e.g., diiron-centre oxygenases, lipoxygenases, peroxygenases, they are less effective. The main problem with chemoenzymatic epoxidation at large-scale is the deactivation of the expensive lipase B enzyme. The deactivation is particularly effective at elevated concentrations of hydrogen peroxide, too high temperatures and in the presence of alcohols, especially ethanol. The effective working time of the catalyst also depends on other process parameters, so the impact of technological parameters on the time of lipase use has been also studied. The rate of epoxidation increases with increasing process temperature, and in a certain range of initial hydrogen peroxide concentrations (10–50 wt %) and amount of lipase. Other important parameters are the amount of solvent (usually toluene), molar ratio of hydrogen peroxide to unsaturated bonds, type of oil or unsaturated acid or alkyl ester of unsaturated acid. In contrast to the classical epoxidation processes based on the use of peroxy acids, in the enzymatic processes the reduction in yield is a result of a lower conversion (lower degree of transformation of unsaturated bonds). It is not a consequence of a lower selectivity transformation of unsaturated bonds to epoxy compounds. The epoxide ring opening side reactions do not occur in the process or they are of minor importance. The reason is that in chemoenzymatic epoxidation there is no need to use strong mineral acids (H_2_SO_4_, HNO_3_, H_3_PO_4_) that catalyse the formation of peroxyacid but at the same time induce oxirane ring opening. The absence of a low-molecular carboxylic acid (acetic or formic) means that no hydroxyesters are formed. Chemoenzymatic epoxidation shows the highest selectivity from among the epoxidation methods mentioned and moreover it is highly stereoselective. In the process of chemoenzymatic epoxidation of vegetable oils, the highest efficiency (also conversion of unsaturated bonds) and the reaction rates were achieved by introduction, prior to epoxidation, of small amounts of a higher carboxylic acid (up to 5% relative to the number of C=C bonds) or a mixture of acids, preferably those already present in the oil.

Due to the enhanced decomposition of concentrated solutions of hydrogen peroxide and a possibility of explosive decomposition of these solutions if their concentration exceeds 60 wt %, water solutions of hydrogen peroxide at a concentration of 30 wt % are usually used. Because of inactivation of lipase B the hydrogen peroxide concentration in the reaction medium usually does not exceed 1 wt %. As yet the highest stability of lipase B has been obtained on epoxidation of unsaturated fatty acids in the presence of peroxyacids generated *in situ*.

The main benefits of chemoenzymatic epoxidation of vegetable oils, unsaturated fatty acids and their alkyl esters include:

- mild reaction conditions, 25–55 °C,
- neutral pH of the reaction mixture,
- possibility of carrying out the process without solvent, which facilitates product separation,
- formation of stable carboxylic peroxyacids under the effect of hydrogen peroxide and in the presence of the enzyme applied; when vegetable oils are used – the above formation also takes place as a result of perhydrolysis,
- possibility of useing immobilized lipase as a biocatalyst (native enzymes can occur in the form of liquids or solid powders),
- high chemo-, region- and stereoselectivity,
- often high conversion of unsaturated bonds in vegetable oils, unsaturated fatty acids and fatty acids esters,
- small contribution or the absence of side reactions (high selectivity),
- the method is safe and environmentally friendly.

The main disadvantage is the high dilution of the reaction solution. Based on the literature review it can be concluded that a large variety of epoxidation methods and technological parameters exist. This study reveals the advantages and distinct efficiency of chemoenzymatic methods. At the same time it allows us to note the need for the individual establishment of technological parameters for a given vegetable oil, unsaturated fatty acid or fatty acid alkyl ester.

**Table 1:** Technological parameters chemoenzymatic epoxidation of vegetable oils, free fatty acids and their alkyl esters

Substrate	Amount of Free Fatty Acid	H_2_O_2_/C=C Molar Ratio mol/mol	Temperature (°C)	Catalyst (wt %)	Solvent	Mixing (rpm)	Reaction Time (h)	Conversion (%)	Reference
Soybean oil	Oleic acid 8 wt %/SO	2:1	50	Lipase B/acrylic resin	Toluene	350	24	95–99	[73]
Soybean oil methyl esters IN = 133.0 g/100 g oil	FFA/SME = 0.001:1 mol/g	1.4 g H_2_O_2_/1 g SME	55	3% Lipase B/acrylic resin	5:1 g/g toluene/esters	800	10–12	98	[72]
Sunflower oil methyl esters	Octanoic acid/esters = 10 mmol/g	not specified	30	Lipase B 10 times was reused	CH_2_Cl_2_, CH_2_Cl_2_-H_2_O	not specified	16	99	[76]
Oleic acid or ethyl oleate	not specified	H_2_O_2_ concentration in solution 0.2 wt %	55	10% Amano lipase from *Burkholderia cepacia* *	Ethyl acetate	150	3	88	[27]
Safindus muko rossi seed oil IN = 84.8 g/100 g oil	Stearic acid	4:1	50	2 wt % Lipase B/oil	Toluene	800	7	90.2	[75]
Rapeseed methyl esters	FFA are formed by ester hydrolysis	H_2_O_2_ concentration in water phase-15 M	40	3 wt % Lipase B/RME	Solvent-free	450	14	83	[85]

*: by weight of oleic acid, SO: soybean oil, IN: iodine number, FFA: free fatty acids, SME: soybean oil methyl ester, RME: rapeseed methyl ester.
